# Plant-biomass-based hybrid seed wraps mitigate yield and post-harvest losses among smallholder farmers in sub-Saharan Africa

**DOI:** 10.1038/s43016-023-00695-z

**Published:** 2023-02-16

**Authors:** Tahira Pirzada, Antoine Affokpon, Richard H. Guenther, Reny Mathew, Sachin Agate, Aitana Blevins, Medwick V. Byrd, Tim L. Sit, Stephen R. Koenning, Eric L. Davis, Lokendra Pal, Charles H. Opperman, Saad A. Khan

**Affiliations:** 1 Department of Chemical and Biomolecular Engineering, North Carolina State University, Raleigh, NC USA; 2 School of Plant Sciences, Faculty of Agronomic Sciences, University of Abomey-Calavi (UAC), Abomey-Calavi, Benin; 3 Department of Entomology and Plant Pathology, North Carolina State University, Raleigh, NC USA; 4 Department of Forest Biomaterials, North Carolina State University, Raleigh, NC USA

**Keywords:** Agriculture, Developing world, Bioinspired materials, Chemical engineering

## Abstract

Sustainable practices that reduce food loss are essential for enhancing global food security. We report a ‘wrap and plant’ seed treatment platform to protect crops from soil-borne pathogens. Developed from the abundantly available wastes of banana harvest and recycled old, corrugated cardboard boxes via chemical-free pulping, these paper-like biodegradable seed wraps exhibit tunable integrity and bioavailability of loaded moieties. These wraps were used for nematode control on yam (*Dioscorea cayenensis-rotundata*) seed pieces in Benin, a major producer of this staple crop in the sub-Saharan African ‘yam belt’. Our seed wraps loaded with ultra-low-volume abamectin (1/100 ≤ commercial formulation) consistently controlled yam nematode (*Scutellonema bradys*) populations while considerably increasing the yield at various locations over 2015–2018. Substantial reduction in post-harvest tuber weight loss and cracking was observed after 3 and 5 months of storage, contributing to increased value, nutrition and stakeholders’ preference for the wrap and plant treatment.

## Abstract (web summary)

Nematode infections of cash crops impact economic well-being and food security in many parts of sub-Saharan Africa. This study reports a nature-based technology developed to protect yam seeds from soil-borne pathogens, increase yield and decrease post-harvest losses among smallholder farmers in Benin.

## Main

The agricultural sector needs to adopt holistic approaches to food production as global food supply must be sustainably enhanced for an exponentially growing population. This is especially relevant in regions dominated by smallholder farming, for example, sub-Saharan Africa (SSA)^1–3^. SSA’s population is expected to increase almost threefold by 2100 (ref. ^3^); however, its agricultural productivity is not keeping pace^4^. This is because of unfavourable climate changes, poor soil fertility, continuous cultivation, pathogen pressure and, to a great extent, knowledge and resource deficiency of smallholder farmers—who represent about 80% of the population^5–9^. Among various tuber and root crops, yam (*Dioscorea* *rotundata* and *D. alata)* is extremely valuable in West Africa, impacting the economic fate of millions of smallholder farmers^10–14^. Various climatic, cultural and edaphic factors dictate yam crop yield and quality^11^; more importantly, 17–50% of the crop is lost annually to plant-parasitic nematodes (PPNs)^13,15–17^.

PPNs, especially root-knot (*Meloidogyne* spp.), yam (*Scutellonema bradys*) and lesion (*Pratylenchus* spp.)^13,14,18^, play major roles in reducing yam crop values primarily because of lack of available, affordable control options^6,9,11^. The yam nematode *S. bradys* is probably the single-most important parasite due to its ubiquitous presence in yam plantings^14^. Being a migratory endoparasite (occurs in roots and tubers, as well as soil) of yam, it is commonly transported regionally with yam seed pieces^18,19^. Its continued reproduction in stored yam leads to the loss of valuable food products and also contributes to the nematode population density at planting because yam seed piece stock comes from infested, stored tubers^13,19,20^. While PPN pre-plant density is negatively correlated with crop yield, in the yam–*Scutellonema* pathosystem initial nematode density often results from nematodes in seed pieces^21,22^ rather than in the soil at planting.

Early PPN protection is critical to crop success. One nematicide application method is via seed treatment, reducing the active ingredient (AI) amount as compared to broadcast or in-row applications, exposing the PPN to a higher AI concentration only in the crop root zone, and substantially reducing non-target effects and environmental impact^23^. Exploring various options for a cost-effective, biodegradable delivery agent, our previous studies indicate promising attributes of paper-like matrices developed via a chemical-free route from banana (*Musa acuminata*) harvest waste as a platform for controlled, targeted delivery of small molecules^8,24,25^. Besides its abundance, cost-effectiveness, composition and durability^24,26–29^, the unique three-dimensional open porous hierarchical structure and comparatively low density make banana fibre (BF) an ideal substrate for loading small molecules. Here, we present a unique ‘wrap and plant’ (W&P) platform to sustainably generate controlled-release biodegradable matrices as seed/seed piece wraps. To fine-tune BF strength and release profile, we utilized recycled, old, corrugated cardboard (OCC) packaging materials developed from one of the most consumed types of paper—paperboard^30–35^. After determining an appropriate composition, we utilized these matrices with and without ultra-low volumes of a nematicide abamectin (Abm) as yam seed wraps in multiple field trials in three different Benin districts from 2015 to 2018. Benin is one of five major yam-producing West African countries (the ‘yam belt’) that produce ~92% of yams globally^10^. Assuming early season control could translate into reduced post-harvest losses primarily caused by *S. bradys*^12,22,36,37^, we also evaluated tuber quality in terms of *S. bradys* reproduction factor, tuber weight, and dry rot and cracking extent after 3 and 5 months of storage.

## Results and discussion

### Structure and composition of BF, OCC and hybrid matrices

**Fig. 1:**
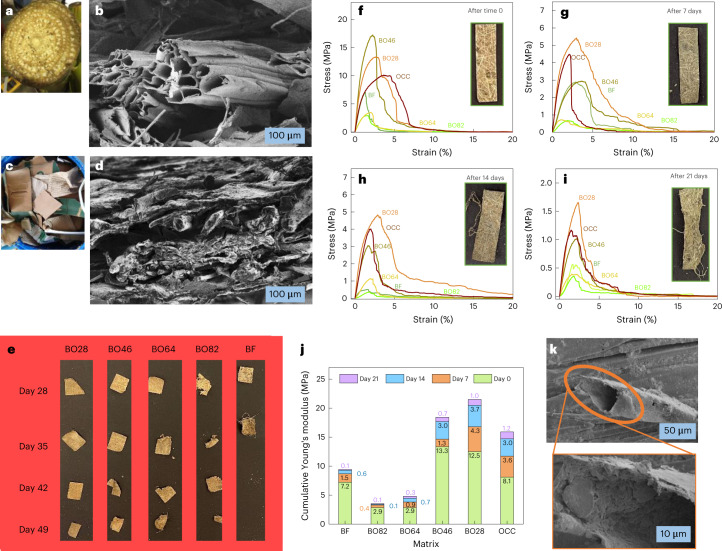
Structure of raw BF and OCC, and mechanical strength of them and their hybrids in the soil. **a**,**b**, Photograph of banana pseudostem (**a**) and representative SEM cross-sections of BF paper (**b**; three samples of different BF papers were scanned at five different magnifications to verify the reproducibility of data). **c**,**d**, Photograph of OCC boxes (**c**) and cross-section SEM of OCC paper (**d**; three samples of different OCC papers were scanned at five different magnifications to verify the reproducibility of data). **e**, Photographs showing pieces of BF and hybrid papers (BO82, BO64, BO46 and BO28) removed from the soil after 4, 5, 6 and 7 weeks. **f**–**i**, Stress–strain plots of paper made from BF, BO82, BO64, BO46, BO28 and OCC before burying in the soil (**f**) and after 1 (**g**), 2 (**h**) and 3 (**i**) weeks of incubation with roots of live tomato plants in soil. Insets show photographs of the corresponding BF after removal from soil. **j**, Plot showing cumulative Young’s moduli of BF, BO82, BO64, BO46, BO28 and OCC papers before and after incubation in soil for 1, 2 and 3 weeks. **k**, High- and low-magnification SEM images showing soil particles coating the surface and the hollow interior of a banana fibre from BF paper buried in soil for 21 days. Source data

We created biodegradable matrices without any chemical additives by simply dewatering a slurry of chopped BF (Fig. 1a,b) and OCC (Fig. 1c,d) individually or mixed in various proportions of BF:OCC as follows: BF, BF only; BO82, 80:20; BO64, 60:40; BO46, 40:60; BO28, 20:80; and OCC, OCC only. The rise in coarseness and distinct fibrous structures with higher BF content in the handsheets (circular sheets with a diameter of 15.2–16.5 cm; Supplementary Fig. 1a) can be attributed to the lack of any harsh chemical treatment that is typically used to dissolve fibres. While the lignin content slightly rises with increasing OCC fraction (Supplementary Fig. 1b), the absence of any additional peaks in the Fourier transform infrared spectroscopy spectra of the hybrids indicates no new functional group is introduced by OCC (Supplementary Fig. 1c).

**Extended Data Fig. 1:**
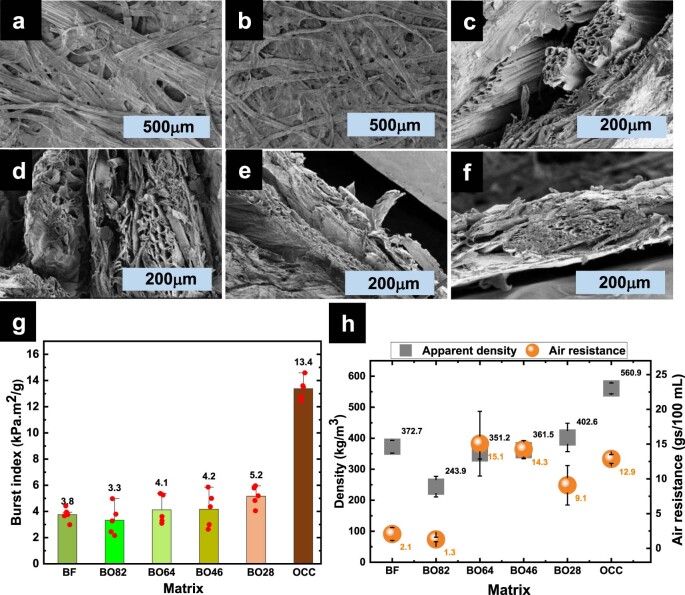
Physical attributes of handsheets made from BF, OCC and hybrid fibers. SEM images showing surface sections of paper made from (a) banana fiber (BF) and (b) OCC. SEM images showing variation in the fiber morphology of the cross sections of the handsheets developed from BF:OCC fibers mixed in various proportions, (c) BO82 (80:20), (d) BO64 (60:40), (e) BO46 (40:60) and (f) BO28 (20:80). 3 independent replicates of each sample were scanned at 5 different magnifications to verify the reproducibility of data. Plots showing variation in (g) burst indices and (h) density and air resistance (inversely related to porosity) of the handsheets prepared from BF, OCC and hybrid fibers (BO). Error bars represent standard deviation in the respective mean values of 10 independent replicates. handsheets. Source data

Scanning electron microscopy (SEM) images of BF (Extended Data Fig. 1a) and OCC (Extended Data Fig. 1b) surface sections show analogous morphology with loosely packed fibres, arising from the similar manufacturing of the handsheets. However, cross-sections of the corresponding handsheets show a sharp contrast in the fibre morphology and packing pattern (Fig. 1b,d). While BF is abundant in loosely packed, hollow fibres, OCC completely lacks hollow tubular fibres and is abundant in layered structures. When combined in different proportions, the resulting hybrids exhibit a gradual loss in the number of hollow banana fibres with increasing OCC content (Extended Data Fig. 1c–f). In addition to its cost-effectiveness and ease of availability, we used OCC with BF to tune the strength and release profile of the hybrid matrices, because the layered morphology contributed by OCC^30,33^ could facilitate bonding and uniformity of BF paper.

While the fibre morphology and composition of the paper can help dictate cargo release properties, the extent of protection provided to the germinating seed without compromising root growth is another critical parameter for selecting the matrix for field trials. Our preliminary investigations have shown the relationship of burst index (minimum amount of pressure required to rupture per gram of paper) with the root penetration profile of the seed wrap^24^. BF paper shows a burst index of 3.8 kPa m^2^ g^−1^ (Extended Data Fig. 1g), almost 3.5 times lower than the burst index of OCC paper (13.4 kPa m^2^ g^−1^). This difference in strength can be attributed to the extent of bonding due to the packing and nature of fibres in each of the papers, that is, more compact fibres enhance hydrogen bonding in OCC paper, while loosely packed, hollow fibres reduce the strength of BF paper^38–40^. Interestingly, with addition of BF, the strength of BO hybrids decreases substantially, for example, there is a 61% reduction in OCC burst index after adding 20% BF in BO28, indicating the role of comparatively weaker, hollow banana fibres in reducing the strength of OCC fibres. An interplay of morphology–strength–cargo release together with biodegradability dictates the ultimate performance and fate of a W&P matrix in a field environment.

### Biodegradability and soil integrity

**Extended Data Fig. 2:**
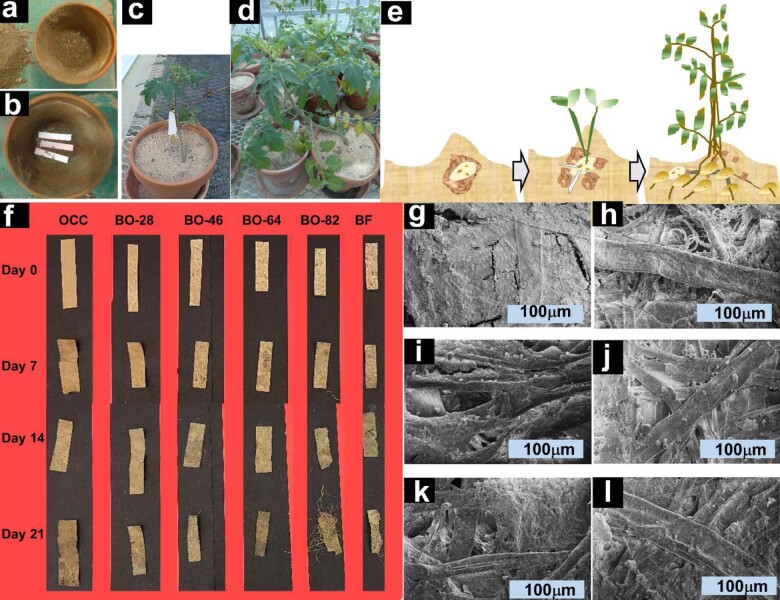
Biodegradability and soil integrity. (a-d) Photographs showing the design of soil integrity studies in the greenhouse using tomato plant as a bioindicator. (e) Schematic proposing the design of ‘W&P’ test based on soil integrity studies. (f) Photographs showing various samples before and after keeping in the soil for 7, 14 and 21 days. SEM images showing the surface section of (g) BF, (h) BO82, (i) BO64, (j) BO46, (k) BO28 and (l) OCC after removing from the soil after 21 days. Red arrowheads show spores of soil micro/macro-organisms. Typically, 3 independent replicates of each sample were scanned at 5 different magnifications to verify the reproducibility of data.

We evaluated strength and integrity of various matrices by incubating them in soil with a growing tomato plant for the period under study (Extended Data Fig. 2a–e). Please note that tomato was only used as a simple bio-indicator of the ability of plant roots to penetrate through various paper compositions. Higher-BF-content papers (BF, BO82, BO64) started losing their strength (Fig. 1f–j, and Extended Data Fig. 2f) within the first three weeks, with a distinct loss in the fibre integrity and strength when kept in the soil for longer periods (Fig. 1e–k and Supplementary Table 1). Lower value of Young’s modulus of the higher BF content samples (indicative of better flexibility) can be attributed to the larger diameter of the BFs (Fig. 1b)^41^, leading to less area available for fibre bonding compared with those of OCC fibres. SEM images of samples drawn from soil after three weeks (Extended Data Fig. 2g–l) show formation of distinct cracks and loss in fibre morphology. BO82 and BO64 hint as a viable wrap that gradually loses its integrity in soil while staying intact during the early growth period. In contrast, paper developed from BF-only disintegrates quickly. We also noticed that even the interior of the hollow banana fibre is coated with soil particles after three weeks (Fig. 1k). This coating can further speed up the degradation process owing to the large surface area available to soil microorganisms. Interestingly, we also noticed spore-like structures on BF, BO82 and BO64 after three weeks (shown as arrowheads in Extended Data Fig. 2g–i), which indicate the supportive environment provided by BF for growth of soil microorganisms that can facilitate biodegradation of BF-enriched matrices in soil. While various studies have recently indicated that decomposition of lignocellulosics in the soil is a synergistic action of several parameters, including diverse enzyme families secreted by different soil microorganisms, composition of lignocellulosics and nature of soil^42,43^, a low lignin content in our synthetic polymer-free BF hybrids taken together with the large surface area (contributed by hollow lumen) exposed to the soil biota can contribute to its biodegradation within a reasonable timeframe.

### Nature and extent of interactions with the AI

**Fig. 2:**
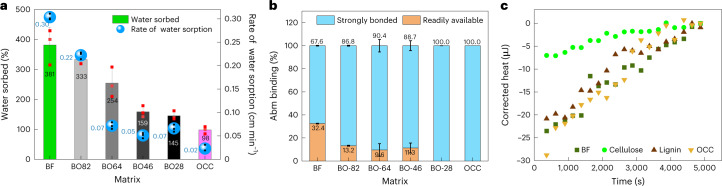
Nature and extent of interactions of BF, OCC and hybrids with water and Abm. **a**, Water sorbed and rate of movement of water by various matrices (*n* = 5, error bars indicate standard deviation from the mean values of amount (%) and rate of water sorbed by BF, OCC and various hybrids). **b**, Strongly and weakly bound Abm content, in BF, OCC and hybrid paper (*n* = 3, error bars represent standard deviation from the mean values of strongly and weakly sorbed Abm on the respective matrices). **c**, ITC thermogram displaying heat released resulting from interactions between Abm with OCC, BF, lignin and cellulose. Source data

The diffusion of small molecules (for example, AI) in a solid phase matrix is dictated by various factors, including interactions between the matrix and diffusant as well as the aqueous pathways (sorption and diffusion) determined by the nature and content of water in the matrix. A higher rate of flow of water in BF and BO82 (Fig. 2a) can be attributed to their low density and high porosity (indicated by low air resistance in Extended Data Fig. 1h) and abundance of hollow tubular fibres acting as a highway for water molecules^44,45^. In addition, thermogravimetric analysis of the matrices shows a large amount of hard-to-remove (bound and trapped^46^) water (56.5 mg g^−1^) and volatiles (15.4 mg g^−1^) per gram in BO82 as compared with high-OCC-content papers, which exhibit a high content of weakly bonded water (free water, indicated by weight loss between 25 and 50 °C; Supplementary Fig. 2a). Generally, hard-to-remove water in a matrix is responsible for diffusion of dissolved/suspended molecules within the fibres, while free water (trapped inside the porous structure^41^) impacts surface phenomena such as adsorption and drying. Comparatively higher content of hard-to-remove water together with faster flow of water in high-BF-content papers (BF and BO82) indicates their tendency to quickly absorb and diffuse small molecules (pesticides, nutrients) dissolved/suspended in water, which facilitates the AI spray and diffusion in the seed wraps.

For longer-term bioavailability to PPNs, the matrix should slowly release AI (here Abm) to ensure continuous exposure to the nematode population, particularly during the early growth period. Also, excess Abm binds with soil, leading to loss of bioavailability^24,25^. As depicted in Fig. 2b, the readily available Abm content decreases with the rise in OCC content in the hybrids, while BF shows the maximum amount (32% of the total load) of readily available Abm. OCC and BO28 papers do not exhibit any detectable weakly bonded Abm, which is not the desired attribute to control PPN populations during the early plant growth periods.

To decouple the interactions of Abm with the components of BF and OCC, and the major building blocks of lignocellulosics (cellulose and lignin), preliminary isothermal titration calorimetry (ITC) measurements were conducted (Fig. 2c and Supplementary Fig. 2b). All experiments show exothermic interactions of Abm with BF, OCC, lignin and cellulose. The ITC thermograms between Abm and OCC and lignin show a similar two-step binding, where an initial steep heat change transitions to smaller heat changes with each titration, indicating saturation of binding sites. Contrarily, BF shows multistep interactions with Abm, showing Abm binding on various sites of BF. When heats of interactions are compared, cellulose releases only 6.9 μJ of heat, which can be assigned to the abundant –OH groups in cellulose leading to weaker interactions with the hydrophobic Abm molecule (structure in Supplementary Fig. 2c). OCC, in contrast, releases ~28 μJ of heat, indicating stronger binding with Abm, while BF and lignin exhibit a similar extent of binding with Abm, as indicated by similar values of heat of interaction of 23 μJ and 21 μJ, respectively. Taken together, the extent of Abm binding and isotherm shape (Fig. 2b,c) indicate that multiple factors are involved in dictating the release profile of these matrices. While a higher OCC content enhances the strength and AI binding of the matrix, BF brings in faster diffusion of dissolved/suspended molecules and appropriate mechanical flexibility needed for a seed wrap. Additionally, the BF hollow fibrous structure increases available surface area for adsorption and diffusion of cargo molecules (AI) dissolved/suspended in water.

### Pilot-scale production of the W&P matrix for field trials

**Fig. 3:**
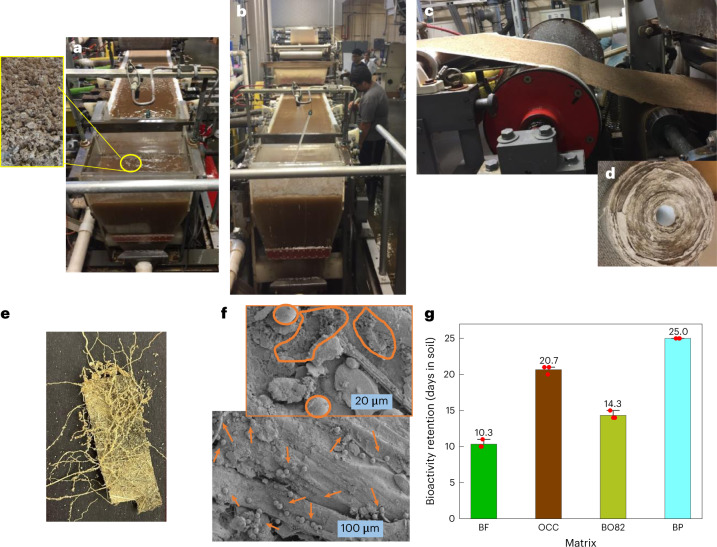
Pilot-scale production and quality evaluation of banana paper. **a**–**d**, Photographs showing paper production on a Fourdrinier machine from a slurry of fibres from the headbox (**a**), which is evenly spread and dewatered to produce wet fibrous sheet on the wire section (**b**) and, on further removal of water in the press and dryer sections (**c**), is converted to a paper roll (**d**). **e**, Photograph of banana paper with tomato roots penetrated after keeping in the soil with a tomato plant for 21 days. **f**, High- and low-magnification SEM images of the surface section of banana paper kept in soil for 28 days. Arrowheads in the bottom micrograph and encircled areas in the top indicate presence of spores of soil microorganisms. **g**, Plot showing data distribution and standard deviation in the mean value of bioavailability of Abm from BF, OCC, BO82 and BP after keeping in soil for 25 days (*n* = 3). Source data

**Extended Data Fig. 3:**
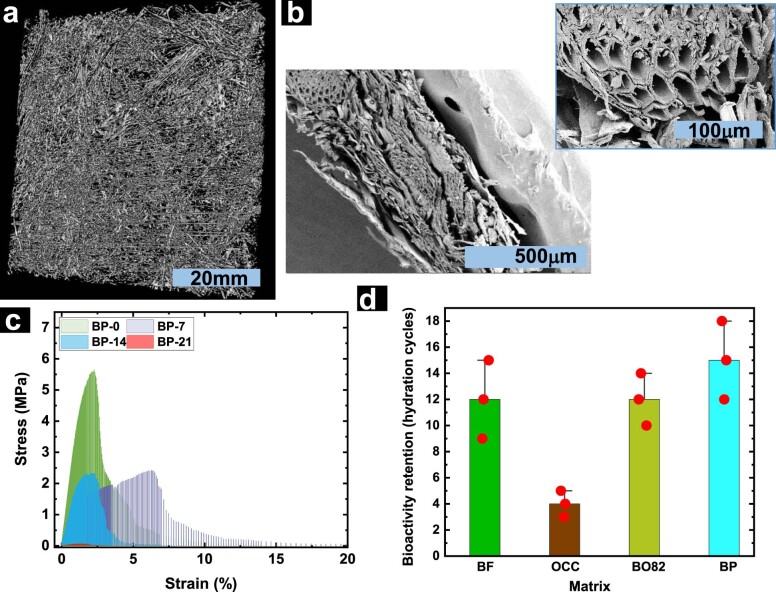
Structure, soil integrity & bioactivity retention of banana paper. (a) X-ray tomograph showing 3D random orientation and loose packing of fibers in banana paper. Two independent replicates of banana paper were scanned at different magnifications and locations to verify the reproducibility of data. (b) High and low magnification SEM images showing morphology of a cross section of banana paper. Three independent replicates of the paper were scanned at 5 different magnifications to verify the reproducibility of data. (c) Stress-strain plot of as-prepared banana paper (BP-0), and after keeping in soil for 7 (BP-7), 14 (BP-14) and 21 days (BP-21). Typically three independent replicates of each sample are tested to verify the reproducibility of data. (d) Plot showing bioavailability of Abm from BF, OCC, BO82 and BP after 15 hydration cycles (n = 3). Error bars show standard deviation in the mean bioavailability data in each case. Source data

Studies conducted in the previous sections indicate BO82 as a suitable candidate in terms of a balance in biodegradability, release, water sorption and strength. For field testing, a 25.4-cm-wide paper roll (referred to as BP) was prepared by dewatering a 0.3–0.5 wt% fibrous slurry consisting of BF and OCC in an 80:20 composition (Fig. 3a–d). The random orientation of the loose-packed fibre and hollow fibrous morphology was verified via X-ray tomogram and SEM micrographs (Extended Data Fig. 3a,b), while burst index, porosity and mechanical performance were optimized to stay within a range similar to BF and BO82 (Extended Data Fig. 3c and Supplementary Table 2). Penetration of tomato roots through an intact paper (Fig. 3e) indicated a balance in strength and root penetration profile. Interestingly, we noticed that BP revealed abundant spores of soil microorganisms after a month in the soil (Fig. 3f). This observation indicates its propensity to biodegrade to low-molecular-weight compounds through the activity of micro- and macroorganisms^47^.

To understand the impact of the soil environment on the bioavailability of AI, we compared the bioactivity of Abm from BP, OCC, BF and BO82 (Fig. 3g) in *Caenorhabditis elegans* bioassays^24,49^, after burying the Abm-loaded matrices in soil for various intervals. The extended retention of bioactivity shown by BP indicates that the W&P formulation partially sequestered the Abm, limiting its interaction with the soil environment. These results, taken together with the water sorption studies demonstrated in the previous section, suggest that as compared with OCC and BF, most of the Abm applied on BP associates with the matrix through the trapped water (hard-to-remove), rather than remaining on the surface, and therefore releases slowly regardless of the hydration cycles (Extended Data Fig. 3d) and soil environment. In addition, our preliminary studies indicate enhanced ultraviolet (UV) stability of otherwise photolytic Abm^48,49^ when loaded on BP (Supplementary Fig. 3), pointing towards the shelf-stable nature of BP–Abm matrix.

### W&P improving the yield of yam crops

**Fig. 4:**
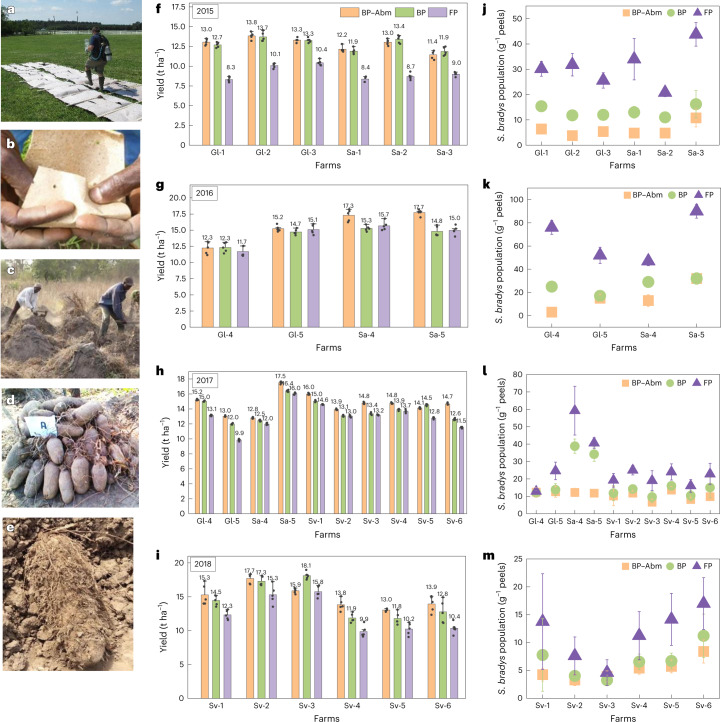
Field trials photographs and data showing yield and *S. bradys* population in tuber peels. **a**–**e**, Photographs showing paper being sprayed with Abm at the North Carolina State University turfgrass site (**a**), a yam seed piece being wrapped in paper (**b**), harvest in yam fields (**c**), healthy tubers obtained after W&P treatment with BP–Abm (**d**) and tubers produced using FP. (**e**). **f**–**i**, Plots showing data distribution and standard deviation in the mean value (shown as individual labels for each bar) in the yield of yam crops in t ha^−1^ in Glazoué (Gl-1, Gl-2, Gl-3, Gl-4, Gl-5), Savè (Sa-1, Sa-2, Sa-3, Sa-4, Sa-5) and Savalou (Sv-1, Sv-2, Sv-3, Sv-4, Sv-5, Sv-6) as a result of field trials conducted in 2015 (**f**), 2016 (**g**), 2017 (**h**) and 2018 (**i**; *n* = 5 for each treatment (FP, BP–Abm and BP) in each field). **j**–**m**, Plots showing population of *S. bradys* per gram of tuber peels at harvest in Glazoué, Savè and Savalou from field trials conducted in 2015 (**j**), 2016 (**k**), 2017 (**l**) and 2018 (**m**; *n* = 3 for peels removed from each treatment (FP, BP–Abm and BP) in each plot). The letters in the acronyms refer to the names of the county, while the numbers indicate the farm number in the respective county, that is, Glazoué, Save and Savalou, which were part of this study. FP = farmers’ practice (no wrap, no Abm); BP = paper only; BP–Abm = Abm-loaded paper. Error bars in all the plots indicate standard deviation from the arithmetic means. Source data

**Extended Data Fig. 4:**
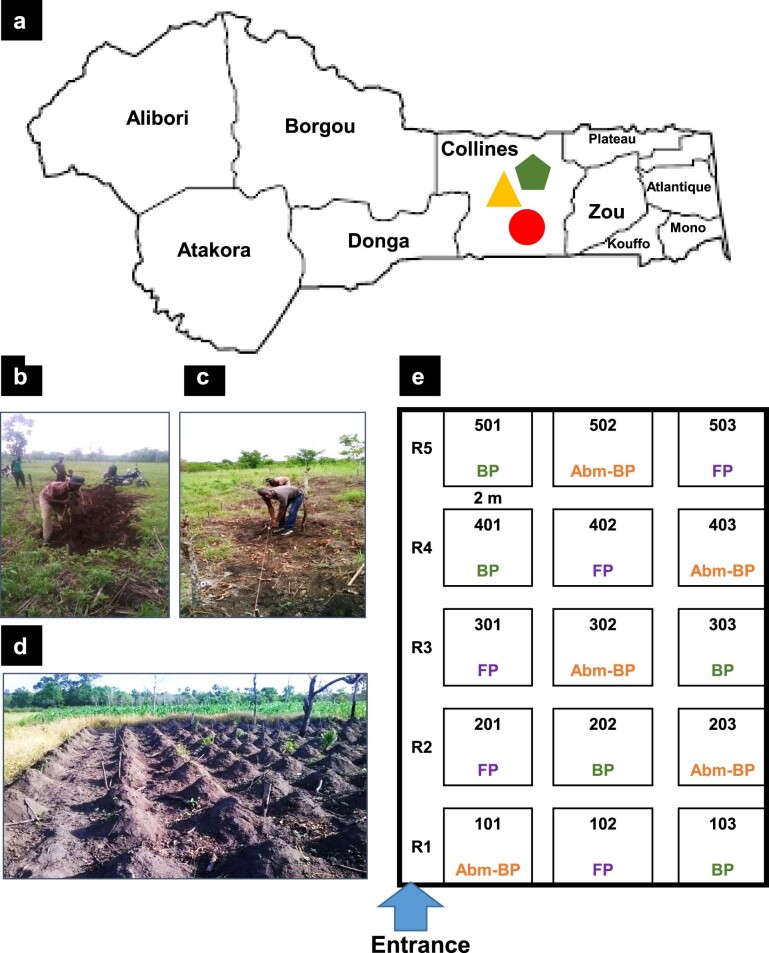
Field trials design & setup. (a) Map showing Savé (green), Glazoué (orange) and Savalou (red) regions in Benin.The base map was applied without endorsement using data from the Database of Global Administrative Areas (GADM; https://gadm.org/). (b-d) Photographs showing preparation and set up of yam fields. (e) Schematic showing randomized complete block design of a field with plots treated with abamectin loaded banana paper (BP-Abm), banana paper only (BP) and untreated control referred as Farmers’ practice (FP).

**Extended Data Fig. 5:**
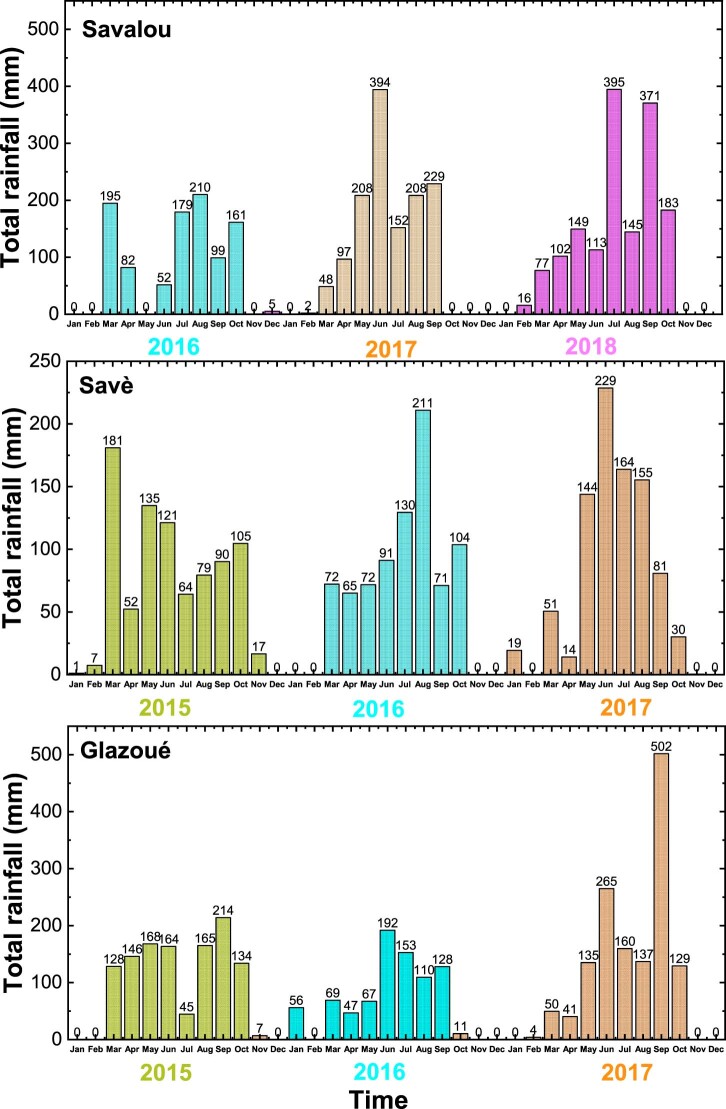
Rainfall patterns in Glazoue, Save and Savalou. Rainfall data for Savé (2015-2017), Galzoué (2015-2017) and Savalou (2017-2018). Source data

We established on-farm field trials in Savè and Glazoué counties in Benin in 2015 (Extended Data Fig. 4). All trials displayed a considerable improvement in tuber weight and quality using W&P treatment compared with conventional farmers’ practice (FP: no BP, no Abm; Fig. 4a–e and Supplementary Video). Figure 4f also displays an impressive increase in yield, that is, 28–57% with Abm-loaded BP (BP–Abm) and 27–53% with untreated BP as seed wraps. The control plots (FP) were the lowest yielding and poorest quality in all cases. In 2016, yield increase was observed for both BP–Abm and BP-only treatments in all trials in Savè and Glazoué regions (Fig. 4g). However, smaller differences are noticed between FP and W&P treatments owing to heavy rains (Extended Data Fig. 5) and flooding of fields. In 2017, we observe a substantial rise in yield of treated (BP–Abm and BP) crops from the same fields in Savè and Glazoué (Fig. 4h). Six Savalou county fields were also included in the study; we notice a 9–22% increase in yield after W&P treatments. In 2018, all six trials conducted in Savalou show a 16–40% rise in the yield as compared with FP (Fig. 4i). Notably, field trials conducted in almost all locations show an overall yield increase from 2015 to 2018, for example, for BP–Abm treatments, yield increased from 13.2 metric tons per hectare (t ha^−1^) in 2015 to 15.2 t ha^−1^ in 2018 with concomitant increase in tuber quality.

Rainfall patterns varied widely during the 4-year study (Extended Data Fig. 5). A general trend was observed that less rainfall led to greater yield differences between treated and FP plots. This was particularly noticeable during the 2015 season (Fig. 3 and Extended Data Fig. 5). Rainfall was much heavier during the 2016–2018 seasons, and in some cases, fields were flooded for a period. For example, heavy rain in August 2016 in Savè led to flooding. In Glazoué, rainfall in 2016 was substantially higher than in 2015, and very heavy rains fell just after planting in 2017. In addition, a heavy September rain led to flooding as well (Extended Data Fig. 5). Nematode damage to root systems can interfere with the ability to take up water in the soil, but can often be compensated by supplying excess water. It is likely that the smaller differences in yield between treatments observed during wetter seasons were due to this phenomenon. Rainfall just after planting may have resulted in dilution of the already microdose of Abm carried in the W&P treatment, rendering it less effective over time. Despite the smaller yield increases in wetter years, the improvement in yam quality with the W&P treatments was substantial in all the trials^46,47^.

**Fig. 5:**
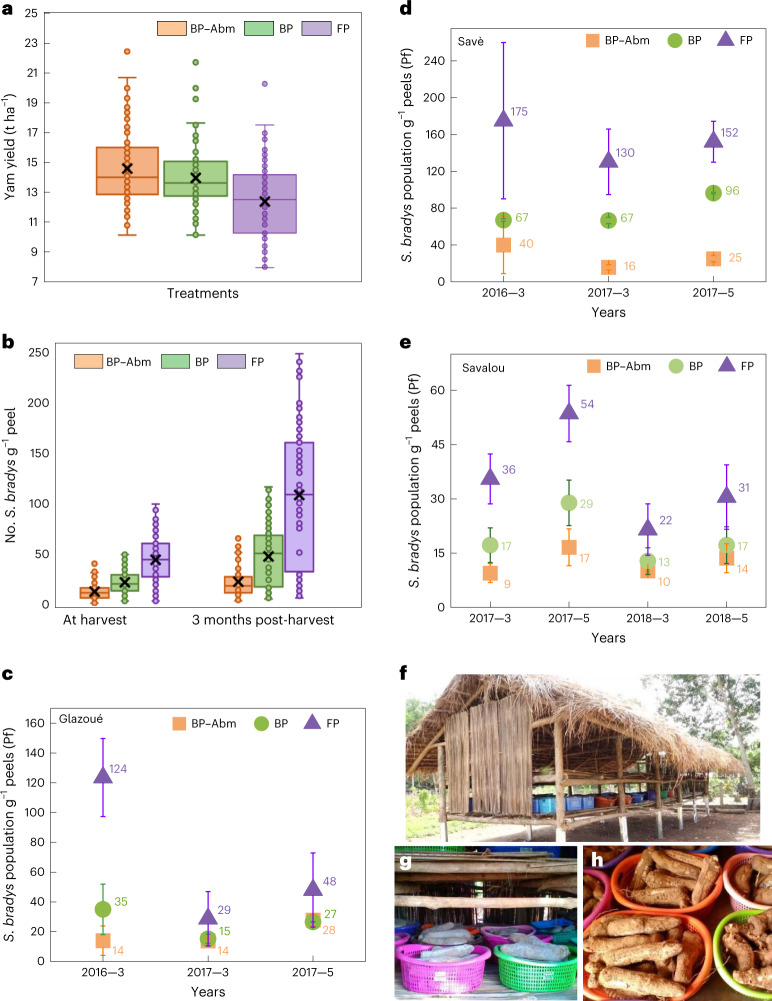
Tuber quality evaluation during storage. **a**, Yam yield in t ha^−1^ as influenced by W&P treatments for 2015–2018 field trials (lsd = 0.34, *α* = 0.01). **b**, Statistical data showing influence of BP–Abm, BP and FP treatments in field trials from 2015 to 2018 on population density of *S. bradys* per gram of yam peel at harvest and 3 months post-harvest (lsd =1.41 and 2.95, respectively, *α* = 0.01). **c**–**e**, Plots showing *S.bradys* final population (Pf) after storing tubers (produced after FP, BP and BP–Abm treatments) for 3 and 5 months in Glazoué (**c**), Savè (**d**) and Savalou (**e**) regions (*n* = 3 for each treatment). Numbers 3 and 5 after each year on the *x*-axis labels indicate the storage time in months. Tubers collected from different sites were stored in the same yam barn, so the storability experiment was conducted in the same climatic conditions to avoid the influence on the results. **f**–**h**, Photographs showing yam tubers stored to evaluate tuber weight, quality and *S. bradys* population (**f**), individually labelled porous containers filled with yam tubers produced after treatment and FP (**g**) and tubers with peels removed for sampling (**h**). Data analysis in panels **a** and **b** consists of one-way ANOVA for a randomized complete block design with three treatments and five replications. Combined analysis was done as for a factorial design with three treatments (BP–Abm, BP and FP), five replications, 26 farms and 4 years with no adjustments. All data analysis was accomplished using the general linear models procedure of PC/SAS software (SAS Institute). Mean separation was done by Waller–Duncan *k*-ratio *t*-test. Boxes are bounded by quartile 1 (bottom, 25th percentile) and quartile 3 (75th percentile), whiskers as minimum (quartile 1 − 1.5 × interquartile range) and maximum (quartile 3 + 1.5 × interquartile range), median defined by the line in the box (interquartile range), mean depicted by X and outliers depicted by dots. Error bars in panels **a**–**e** indicate standard deviation from the arithmetic means (shown as respective labels). Source data

**Extended Data Fig. 6:**
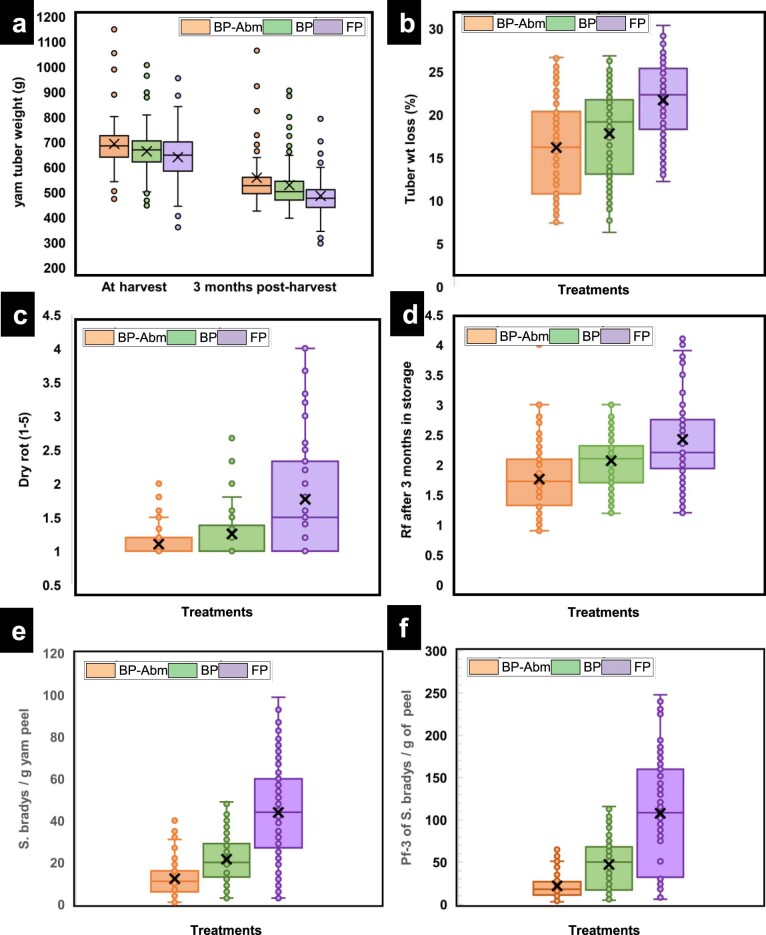
Effect of treatments on yield & tuber storage. Data showing (a) Yam tuber wt (g / tuber) at harvest and 3 months post-harvest (least significant difference (lsd) 31.2 and 26.5 respectively α = 0.01), (b) percent weight loss of tubers after 3 months storage. (lsd = 0.81, α = 0.01), (c) Yam dry rot on a 1- 5 scale as influenced by W&P treatments (lsd = 0.10, alpha 0.01)., and (d) 3 months post-harvest reproductive factor (Rf 3 = population density at 3 months / initial {harvest} population density / g of yam peel) for Scutellonema bradys (lsd =0.12, α = 0.01), (e) Influence of W&P treatments on final population density of Scutellonema bradys per gram of yam peal from 2015-2018 (least significant difference =1.41,α = 0.01), and (f) Population density (Pf3/g of yam peel) 3 months post harvest for Scutellonema bradys as affected by W&P treatments from 2016 – 2018 (lsd = 2.95, α = 0.01). Data analysis consisted of one-way Analysis of Variance (ANOVA) for a randomized complete block design with three treatments and five replications. Combined analysis was done as for a factorial design with three treatments (BP-Abm, BP and FP), five replications, 26 farms, and four years with no adjustments. All data analysis was accomplished using the General linear models procedure (PROC GLM) of PC/SAS software (SAS Institute, Cary, NC). Mean separation was by Waller-Duncan K-ratio t-test. Boxes are bounded by Q1 (bottom, 25th percentile) and Q3 (75th percentile), whiskers as minimum (Q1-1.5*IQR) and maximum (Q3 + 1.5*IQR), median defined by line in the box (IQR), mean depicted by X, outliers depicted by dots. Source data

Statistical analysis of data (Extended Data Fig. 6 and Supplementary Table 3) indicates that yam yields were increased in all years (*P* ≤ 0.01) by W&P treatments compared with FP (Fig. 5a). Although BP–Abm was generally superior to untreated paper (BP), the difference between the two was often indistinguishable (Extended Data Fig. 6a,b), suggesting that BP alone provided a distinct advantage over FP. Previous studies have demonstrated that BP interferes with host–nematode chemical communications by binding host root exudates and impacting the nematode’s ability to locate the root^10^. Dry rot was greater (*P* ≤ 0.01) in FP treatments than in W&P (Extended Data Fig. 6c). The difference in tuber quality (weight, size and health) is noteworthy here; farmers indicated that BP–Abm treatment always resulted in longer and larger tubers that looked free from PPN infection (Fig. 4d,e). Across all field trials, a considerable reduction in yam nematode populations was observed in tuber peels as compared with FP (Fig. 4j–m). When compared with BP-alone and controls (FP), BP–Abm treatment at planting resulted in considerable reductions in the final nematode populations. We observed an 80% reduction in final yam nematode populations in tuber peels (Fig. 4j–m). Not only was this largely responsible for the high tuber quality, it also indicates substantially reduced risks of post-harvest tuber damage and loss due to this nematode. Variation in final nematode populations was consistently low across trials during all 4 years of field trials.

### Storage longevity of yam tubers

Yam is also considered an income-generating cash crop because it can be traded, stored and consumed during the ‘hungry months’ when production of other crops is not possible^11^. Healthy tubers from stored yams are also used as seeds for the next season’s crop. Maintaining tuber health post-harvest is a critical challenge, with around 1 million tons of yam being lost annually during storage^50^. While various factors play a role in post-harvest loss, the yam nematode is considered a major contributor, causing dry rot as well as predisposing the tubers to fungal and bacterial infections^14,20^.

Yam tuber weight at harvest was increased by W&P treatments (Fig. 5a; *P* ≤ 0.01) from 2016 to 2018 with substantial improvement over FP (least significant different (lsd) = 31.2, *α* = 0.01). After 3 months’ storage, the tuber weight decreased, with average tuber weights of treatments BP–Abm > BP > FP (lsd = 26.5, *α* = 0.01). The percent weight loss of tubers was affected by W&P treatments (Extended Data Fig. 6a,b; *P* ≤ 0.01). Tubers from FP lost a greater percentage of weight compared with BP–Abm treatments, while BP weight loss percentage was greater than BP–Abm (lsd = 0.81, *α* = 0.01).

In all three regions, we noticed a considerable reduction in *S. bradys* final population in the treated (BP–Abm and BP-alone) tuber peels as compared with FP (Fig. 5b). Statistical analysis of the tuber storage data indicates that W&P treatments resulted in lower (*P* ≤ 0.01) *S. bradys* population density per gram of peels at harvest than FP (Fig. 5b and Supplementary Table 4). The BP–Abm treatment had noticeably lower nematode population densities than untreated BP, which was also lower than FP (lsd = 1.41, *α* = 0.01). While reproduction factor and numbers of *S. bradys* in yam tuber peels increased in tubers after storage (Fig. 5b–e), treatment differences in population density were still present (*P* ≤ 0.01). FP had greater numbers than BP treatments, which in turn was larger than BP–Abm treatment (lsd = 2.95, *α* = 0.01).

**Extended Data Fig. 7:**
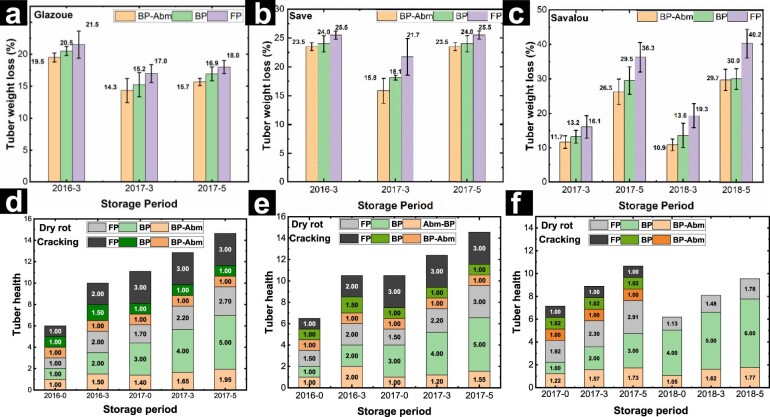
Post-harvest tuber quality evaluation. Post-harvest data showing tuber weight loss and tuber health (dry rot and cracking) after 3- and 5- month storage in (a) & (d) Glazoué (2016-2017), (b) & (e) Savé (2016-2017) and (c) & (f) Savalou (2017-2018) regions. Error bars in part a-c indicate standard deviation from the arithmetic means. Source data

After 5 months of storage, a 2.5–3-fold multiplication in *S. bradys* population was observed in tubers produced from FP treatment, while the population multiplication was between 1.5- and 2-fold for the tubers produced with BP–Abm treatment, placed under similar conditions and durations (Fig. 5c–e). Combined with the lower starting populations in W&P-treated tubers at storage commencement, this is an impressive reduction in the yam nematode burden. Tuber weight loss during storage can be partially associated with moisture loss from tubers drying at high ambient temperatures (25–30 °C). However, the major contributor to yam tuber weight loss during storage is *S. bradys*, which compromises the quality, food value and marketability of the tubers to a large extent. Compared with controls (FP), treated tubers show less weight loss in storage for 3 and 5 months (Extended Data Figs. 6a–b and 7a–c). We also observe a reduction in dry rot and cracking in the W&P-treated tubers over 3 and 5 months of storage in all three regions (Extended Data Figs. 6c and 7d–f).

### Yam stakeholders’ perception of W&P technology

To evaluate farmers’ perception of the W&P technology and its marketability, three major studies were conducted in Savè, Savolou and Glazoué regions (Supplementary Video). An average cost–profit ratio of 79.3% for yams produced after BP–Abm treatments as compared with 59.93% generated via FP indicates that yam production via W&P treatment is economically profitable in terms of variable and fixed production costs^51^. The net margin of 1,112,080 African Financial Community Francs per hectare (FCFA ha^−1^) generated by BP–Abm treatment is 153.7 % higher than that of FP (723,498 FCFA ha^−1^), while BP-alone-treated yams generated a net margin of 768,515 FCFA ha^−1^. Overall, farmers received a much higher price for W&P treated tubers than for tubers produced by FP, due to higher quality and appearance^51^. What remains to be seen is the cost of commercializing this technology. This is an avenue that is being explored with a commitment to making this technology as inexpensive and accessible as possible for smallholder farmers.

We also evaluated the effect of BP–Abm treatment on the organoleptic qualities of yam tubers by examining attributes such as vegetative, harvest, food processing and palatability of yam tubers produced via W&P treatment^52^. The high appraisal indices for boiled and pounded yam produced via W&P treatment provided strong support by consumers for the adoption of W&P technology for better yam production in West Africa^52^. The response of various stakeholders including farmers, yam-based food processors and traders was recorded based on the vegetative, harvest and tuber processing stages of W&P-treated yam versus FP. In Savalou and Savè, the sensorial/organoleptic quality of foods derived from BP–Abm-treated tubers and the nematological and agronomic aspects represented key components of yam stakeholders’ preference for adoption of this innovation related to yam production. In Glazoué, though, nematological and agro-morphological descriptors were more important in selection of BP for yam production^52^. Overall, the study shows the preference of yam stakeholders from the central region of Benin for the W&P BP–Abm treatment, based on the quality of the tubers and derived foods (for example, flour).

In summary, we have developed a robust, flexible platform for sustainable crop protection for smallholder farmers by recycling wastes from banana harvest and old, corrugated cardboard boxes via a straightforward, chemical-free route. Field trials in three different regions of Benin showed a considerable increase in yield and quality of crops produced from tubers treated with either Abm-loaded or untreated banana paper. Considerable reductions in yam nematode (*S. bradys*) reproduction factors in tuber peels after three- and five-month storage periods indicate the effectiveness of the W&P platform in reducing post-harvest loss. Additionally, Abm-loaded banana paper provided the most protection from dry rot and cracking, followed by banana paper alone. On top of extended release, tunable strength, soil integrity and reduced AI photolysis, W&P technology is a solid phase treatment that does not require additional equipment for application. A single treatment with an ultra-low volume of AI (1/100th or less of a commercial formulation) reduces expenses while minimizing non-target effects. Both farmers’ and consumers’ perceptions of W&P technology, food quality and preparation of yam flour revealed a strong preference for Abm–banana-paper-treated yams, with banana paper alone also outperforming farmer’s practice in terms of quality and storage. In the future, this platform will be amenable to structural modification such as pouches, slips and seedling trays for scalability, ease of application and AI delivery. The tunable nature of our seed wraps is also a promising feature for delivering other crop production moieties, such as macro- and micronutrients, biologicals, or insecticides and fungicides, with application to smallholder farms, organic producers and potentially larger-scale producers.

## Methods

Banana (*Musa acuminate*) fibre was obtained from the Agricultural Industrial Unit of Earth University, Costa Rica. OCC boxes were procured from the North Carolina state’s paper pilot plant. Abm (97%) was supplied by Alfa Aesar. *Caenorhabditis elegans* strain N2 (wild type) were obtained from the Caenorhabditis Genetics Center. Reagent-grade acetone (99.5%) and high-performance liquid chromatography (HPLC)-grade acetonitrile (99.8%) were purchased from Millipore Sigma. Rhodamine B (≥95%) dye was purchased from Millipore Sigma and used without further purification. For lignin content measurement, sodium thiosulfate solution (0.2 N), potassium iodide solution (1 N), sulfuric acid (4 N), potassium permanganate solution (0.1 N) and starch indicator were provided by Fisher Scientific. Deionized (DI) water (pH 5.77 ± 0.13) was used throughout the experiments, except while making paper and spraying for field trials.

To prepare handsheets from BF, OCC and hybrid fibre slurries, the fibres were soaked in water separately or in various compositions (Supplementary Table 1) overnight and diluted to 1.57% consistency before beating in the valley beater for 3 min, as per Technical Association of the Pulp and Paper Industry (TAPPI) T200 standard method. A standard laboratory British handsheet mould was used to prepare at least ten circular handsheets per composition (15.88-cm diameter with a grammage of 70 g m^−^^2^) from the pulp, following TAPPI T205 standard method. After selecting the final composition for the W&P matrix, OCC and BF were mixed and made into a 1.57% consistency pulp slurry by mechanically chopping down (refining) the fibres in water for 3 min in a valley beater. A 30.48-cm Fourdrinier paper machine was used to prepare a 25.4-30.48 cm-wide paper roll (Fig. 3a–d). Prepared fibre slurry was stored in the tank overnight. This slurry was then transformed into a sheet via an open headbox (Fig. 3a) to evenly distribute the suspension onto the wire section for initial dewatering (Fig. 3b). Water was further removed from the wet web in the press section, followed by drying by heated cylinders in the drying section, similar to the process in industrial scale paper machines. The paper was continuously wound on a roll (Fig. 3d). The target grammage (weight per surface area) was 70–80 g m^−^^2^. The paper (handsheets and paper roll) was conditioned at 23 °C and relative humidity of 50% before testing the density, air resistance (Gurley method), burst strength and lignin content, following TAPPI T410, T460, T810 and T236 tests, respectively.

A field emission scanning electron microscope (Verios FE1) was used to characterize the morphology of various samples, which were made conductive by coating with a 10 nm layer of gold before characterization, while the acceleration voltage was kept at 2.0 kV. Typically three cross-sections of different samples of each type of paper were scanned at five different magnifications to verify the reproducibility of data. Fourier transform infrared spectroscopy data were collected using a Perkin Elmer Frontier spectrometer, equipped with a diamond/ZnSe attenuated total reflection stage. Sixty-four scans were performed in each case while the data were accessed via OMNIC software. A TA Instruments SDT thermogravimetric analyzer was used to measure free and hard-to-remove water content of each sample via thermogravimetric analysis. A 10–12 mg sample was heated at a rate of 10 °C min^−1^ to determine the weight loss at 50, 90 and 120 °C. Mechanical performance of samples was examined using a dynamic mechanical analysis attachment on a TA Instruments Discovery Series Hybrid Rheometer HR-3. Rectangular specimens with a length of 30 mm and a width of 10 mm were clamped at the two ends with a loading gap fixed at 15 mm and a constant pulling rate of 3 mm min^−1^. The minimal force for recording to remove any slack of the samples was set at 0.1 N. The thickness of each sample was measured by a micrometre screw gauge. At least three specimens from each sample were evaluated and averaged for all samples.

### Binding studies

To estimate the extent of binding between the matrices and AI (Abm in this case), we sonicated a 1 cm^2^ piece of matrix loaded with 10 ppm of Abm in 10 ml of DI water for 5 min. The same piece of paper was later transferred to 10 ml of acetone and vigorously shaken for 30 min to ensure complete dissolution of Abm. HPLC analysis of the aliquots gave us an estimate for the weakly bound AI released in DI water in the first 5 min and strongly adhered Abm that was collected in acetone. All experiments were conducted in triplicate. To understand the nature of interactions between Abm and components of hybrid matrices (BF and OCC) and their building blocks (lignin and cellulose), ITC was conducted via a TA Instruments isothermal titration calorimeter (NanoITC, TA Instruments) at 298 K. A 2.5 × 10^−^^6 ^M Abm solution in DI:acetone (95:5 ratio by volume) solvent was titrated into a 170 ml cell initially containing the titrant (BF, OCC, lignin or cellulose) dispersions (0.001 wt%) in the same solvent (DI:acetone) via 20 injections of 2.45 µl each. The injections were pre-programmed and automatically carried out at 250 s intervals under 350 rpm stirring rate. The time interval between injections was chosen to ensure that thermodynamic equilibrium was achieved before the next injection. The baseline before the first injection and after the final was collected for 100 s. Abm solution dilution into the solvent (DI:acetone in 95:5 ratio by volume) experiment was performed to collect data regarding the heat of dilution of the titrant. The dependence of the heat of interaction on the ratio of Abm:titrant was obtained by calculating the area of the peaks obtained on each injection. Each titration experiment was performed in triplicate.

### UV stability analysis

An INTELLI-RAY 400 UV lamp with an intensity of 50 mW cm^−^^2^ was used to expose three replicates of Abm only (on copy paper), BP–Abm, Abm–BF and Abm–OCC samples to UV light for 30, 60, 120 and 180 min (Supplementary Fig. 3). Abm content was kept at around 10 ppm per gram of sample. The samples were agitated in acetone for 1 hour to ensure complete dissolution of Abm, which was determined via HPLC.

### Experiment sites

The field study was undertaken from 2015 to 2018 in the Guinea–Sudan transition zone of Benin (centre of Benin, West Africa) in three districts: Glazoué (site of Houin), Savalou (site of Agbadogo) and Savè (site of Gobé). Experiments were carried out for three consecutive years in a total of 16 farmers’ fields at Glazoué (Gl-1, Gl-2, Gl-3, Gl-4 and Gl-5) and Savè (Sa-1, Sa-2, Sa-3, Sa-4 and Sa-5), and for two consecutive years at Savalou (Sv-1, Sv-2, Sv-3, Sv-4, Sv-5 and Sv-6). The acronyms refer to the names of the various farms that were part of this study. The climate where the trials were conducted is tropical Guinea–Sudan humid savannah, with a transitional regime between a bimodal rainfall distribution (southern Benin) and unimodal rainfall distribution (northern Benin). The average annual rainfall in the transition zone is between 900 and 1,200 mm with seasonal variations and unequal distribution, exacerbated by increasing climate change. Most soils in this region are classified as tropical ferruginous soils^53^ with a sandy loam texture (Supplementary Table 5). The sites of Agbadogo and Gobé are located on lowland (moderately well-drained soils), while Houin is located on a plateau (well-drained soils).

### Experiment details and field design

Field activities were carried out in the sub-humid savannah agro-ecological zone, one of the major yam production regions of Benin (Extended Data Fig. 4a). Soil samples were taken for preliminary assessment of nematode infestation levels. After sample analysis, the field with the highest nematode population density was selected. A total of ten fields (five per location) were therefore selected for the trial. Fields were cleared of vegetation, and mounds were made following yam cultivation practices. The mounds were labelled according to the experiment design (Extended Data Fig. 4b–d). Each field trial was arranged in a randomized complete block design with five replicates and three treatments (Extended Data Fig. 4e): plots, spaced 2 m apart, accommodated four rows of six mounds. In each field, trial was arranged in a randomized complete block design with five replicates and three treatments (Extended Data Fig. 4d): (1) banana paper + Abm (BP–Abm); (2) banana paper alone (BP); and (3) untreated control (referred to as farmers’ practice, FP), where seed yams were planted without banana paper. Each BP–Abm wrap (15.25 × 20.25 cm^2^) contained 10 µg of Abm. This resulted in plots with Abm at the rate of 1.0 g ha^−1^ or 4.54 ppb.

After the mounds were prepared for planting and labelled following the treatments, soil samples were collected to estimate pre-planting nematode population densities. Four soil cores were removed per plot from 5 to 30 cm depth using a hand trowel, following a zig-zag sampling pattern^54^. Soil cores from the same plot were combined and thoroughly mixed before a 250 cm^3^ composite sample was removed for nematode extraction using the centrifugation technique described in ref. ^55^. PPNs recovered in the fields were at least one of the three most important genera: *Scutellonema* spp., *Meloidogyne* spp. and *Pratylenchus* spp. Fields harboured an initial nematode density of at least 500 nematodes per 250 cm^3^ soil.

Each mound was planted with single seed yam (cv. Klatchi of the complex *Dioscorea cayenensis-rotundata*) wrapped or not with banana paper, depending on the treatment. Seed yams for the first year of trials were produced in nematode-free soil using miniset technique^56^. For the following years, seed yams were purchased from farmers who had been previously trained for the production of nematode-free seed yams. Briefly, they set up the plantation of treated mother seed yam, then harvested tubers from July to September and kept the vines on the mounds. Small tubers generated by the roots of these vines from September to December were used as clean seed yams for the following years. No fertilizers were applied, and planting and other cultural operations were performed by farmers according to local practices. Planting occurred at the beginning of the first rainy season (8–11 June 2015, 13–16 May 2016, 26 April–29 May 2017, 8 May 2018) and tubers were harvested 7 to 8 months later when vines were completely dried. Typical FP is to plant the yam seed piece in the mound with no further inputs. FPs vary among farms, but weed control is via manual cultivation when it does occur, and no fertilizer is added to the mounds. No insect or disease control agrichemical applications are employed. Yam cultivation is very low input and labour intensive, with crop management being performed manually.

### Tuber yields at harvest and weight loss after storage

Evaluation of post-harvest efficacy of W&P technology was initiated in 2016 to study the effect of treatments on nematode population increase, yam tuber weight decline and quality after storage. Tubers were harvested and yields were determined as the cumulative weights from all mounds and expressed as grams per square metre. The effect of W&P on tuber storability was assessed after three- and five-month storage periods. Two tubers were randomly collected from individual plots at harvest, arranged in plastic bags and further stored in a covered, open-sided yam barn (Fig. 5f) to assess nematode population build-up and tuber damage. Weights of two tubers per plot used for post-harvest studies were recorded before and after storage, and the percentage weight loss was calculated as follows:

$$\begin{array}{l}{{{\mathrm{\% }}}}\;{{{\mathrm{weight}}}}{{{\mathrm{}}}}\;{{{\mathrm{loss}}}} = \\\frac{{100 \times ({{{\mathrm{Mean}}}}\;{{{\mathrm{weight}}}}\;{{{\mathrm{of}}}}\;{{{\mathrm{yam}}}}\;{{{\mathrm{tubers}}}}\;{{{\mathrm{at}}}}\;{{{\mathrm{harvest - Mean}}}}\;{{{\mathrm{weight}}}}\;{{{\mathrm{of}}}}\;{{{\mathrm{yam}}}}\;{{{\mathrm{tubers}}}}\;{{{\mathrm{after}}}}\;{{{\mathrm{storage}}}})}}{{{{{\mathrm{Mean}}}}\;{{{\mathrm{weight}}}}\;{{{\mathrm{of}}}}\;{{{\mathrm{yam}}}}\;{{{\mathrm{tubers}}}}\;{{{\mathrm{at}}}}\;{{{\mathrm{harvest}}}}}}\end{array}$$

Tubers collected from different sites were stored in the same yam barn, so the storability experiment was conducted in the same climatic conditions to avoid the influence on the results. The site of the storability experiment is located in the sub-humid savannah region with a sub-equatorial climate characterized by two wet seasons from mid-March to mid-July and mid-September to mid-November, alternating with two dry seasons. The annual average rainfall is between 1,000 and 1,200 mm, and the yearly mean temperature between 25 and 30 °C.

### Assessment of nematode population densities

Nematode population densities were estimated at harvest from tubers and soil samples. To determine tuber nematode population densities, sample peels (outer cortex) were removed from a 5 × 5 cm^2^ area on four sides of each of three tubers using a kitchen peeler (modified from ref. ^18^; Fig. 5g,h and Supplementary Video). Tuber peels from the same plot were then thoroughly mixed, and 25 g peels were removed for nematode extraction using the centrifugation technique. For soil nematodes, soil sub-samples were collected from the tuber zones in the middle mounds, and a composite soil sample of 250 cm^3^ was removed per plot and processed for population estimation. Thereafter, nematodes were morphologically identified and counted under an Olympus CX31 dissecting microscope at a magnification of ×20.

Nematodes in stored tubers were also extracted from 25 g of yam peels following the procedure described in the previous paragraph. The build-up in *S. bradys* population during storage was determined at yam harvest and after storage. The reproduction factor (Rf) for *S. bradys* during storage was then calculated as:

2 $${{{\mathrm{Rf}}}} = \frac{{{\mathrm{Nematode}}\;{\mathrm{population}}\;{\mathrm{at}}\;{\mathrm{storage}}}}{{{\mathrm{Nematode}}\;{\mathrm{population}}\;{\mathrm{at}}\;{\mathrm{harvest}}}}$$

### Assessment of nematode damage

Prior to peel removal, tuber samples were subjected to visual assessment of nematode symptoms at harvest and after 3 or 5 months of storage. Severity of dry rot and cracking symptoms was rated on a 1–5 scale as follows: 1 = clean tuber; 2 = 1–25% of tuber skin showing targeted symptoms (low level of damage); 3 = 26–50% of tuber skin showing targeted symptoms (low to moderate level of damage); 4 = 51–75% of tuber skin showing targeted symptoms (moderate to severe level of damage); and 5 = 76–100% tuber skin showing the targeted symptoms (high level of damage)^28,57,58^.

### Data analyses

Data analysis for each site consisted of analysis of variance (ANOVA) for a randomized complete block design with three treatments (FP, BP and BP–Abm) and five replications. The yearly results were combined to determine the differences between years. All data analysis was done using the general linear models procedure of PC/SAS software (SAS Institute). The lsd was used for mean separation. Because we observed substantial differences in rainfall patterns and growing practices between farms and years, each year and data between farms within each year were analysed separately.

### Reporting summary

Further information on research design is available in the Nature Portfolio Reporting Summary linked to this article.

## Supplementary information

Supplementary Information Supplementary Discussion, Supplementary Figs. 1–3, Supplementary Tables 1–5 and references.

Reporting Summary

Supplementary Video Video showing setting up and harvesting field trials in Benin.

Supplementary Data Excel file showing source data for the graphs shown in Supplementary Figs. 1–3.

## Data Availability

All data generated or analysed during this study are included in this published article, as separate source data files and Supplementary Information files. Source data are provided with this paper.

## Source data

Source Data Fig. 1 Source data for stress–strain and Young’s modulus plots.
Source Data Fig. 2 Source data for water sorption, abamectin binding and isothermal titration results.
Source Data Fig. 3 Source data on bioactivity retention.
Source Data Fig. 4 Source data on yield and nematode population.
Source Data Fig. 5 Source data for all plots.
Source Data Extended Data Fig. 1 Raw data for burst index, density and air resistance.
Source Data Extended Data Fig. 3 Raw data used to plot stress and bioactivity retentions.
Source Data Extended Data Fig. 5 Source data for rainfall patterns.
Source Data Extended Fig. 6 Source data to analyse various effects of treatment.
Source Data Extended Fig. 7 Source date on tuber health and percent weight loss during storage.
