# Supplementary material for: Plant-biomass-based hybrid seed wraps mitigate yield and post-harvest losses among smallholder farmers in sub-Saharan Africa
Source: Nat Food. 2023 Feb 16;4(2):148–59. doi: 10.1038/s43016-023-00695-z (PMC10154224; doi:10.1038/s43016-023-00695-z)
Supplement: Supplementary file 1 — Supplementary Discussion, Supplementary Figs. 1–3, Supplementary Tables 1–5 and references. [file 43016_2023_695_MOESM1_ESM.pdf]

nature food

Article

https://doi.org/10.1038/s43016-023-00695-z

Plant-biomass-based hybrid seed wraps mitigate yield and post-harvest losses among smallholder farmers in sub-Saharan Africa

In the format provided by the authors and unedited

Table of Contents

**1.**	**Supplementary Discussion**	2
**2.**	**Supplementary Figures**	
	Supplementary Figure 1. Handsheets made from BF, OCC and hybrid fibers: Appearance & Composition	3
	Supplementary Figure 2. Free & bound water content of matrices, ITC titrations & structure of abamectin	4
	Supplementary Figure 3. Experiment setup and data showing delayed decomposition of Abm loaded on BP	5
**3.**	**Supplementary Tables**	
	Supplementary Tables 1. Biodegradability and soil integrity studies.	6
	Supplementary Tables 2. Physical attributes of various matrices.	7
	Supplementary Tables 3. Analysis of variance (ANOVA) data.	8
	Supplementary Tables 4. Communities soil texture (% sand, silt, clay).	
	Supplementary Tables 5. Effect of W&P treatment on variation in nematode population during storage.	9<br>10
**4.**	**Reference**	11

Supplementary Discussion

It must be noted that the lower nematode numbers, and lower reproductive factors (Rf) are not the result of residual abamectin in tubers. The lower Rfs as indicated with paper wrapped tubers are due to a lower nematode population at harvest in treated tubers than untreated tubers because pesticide residues are not relevant when considering abamectin. Abamectin is a large hydrophobic organic molecule that is not systemic in the plant. The half-life of the abamectin (a biological fermentation product) and its movement in soil are all extremely low.[1] Abamectin in harvested tubers would thus be undetectable and likely absent altogether. The lower nematode numbers associated with abamectin treatment indicate the efficacy of the at-planting treatment in suppressing nematode reproduction.

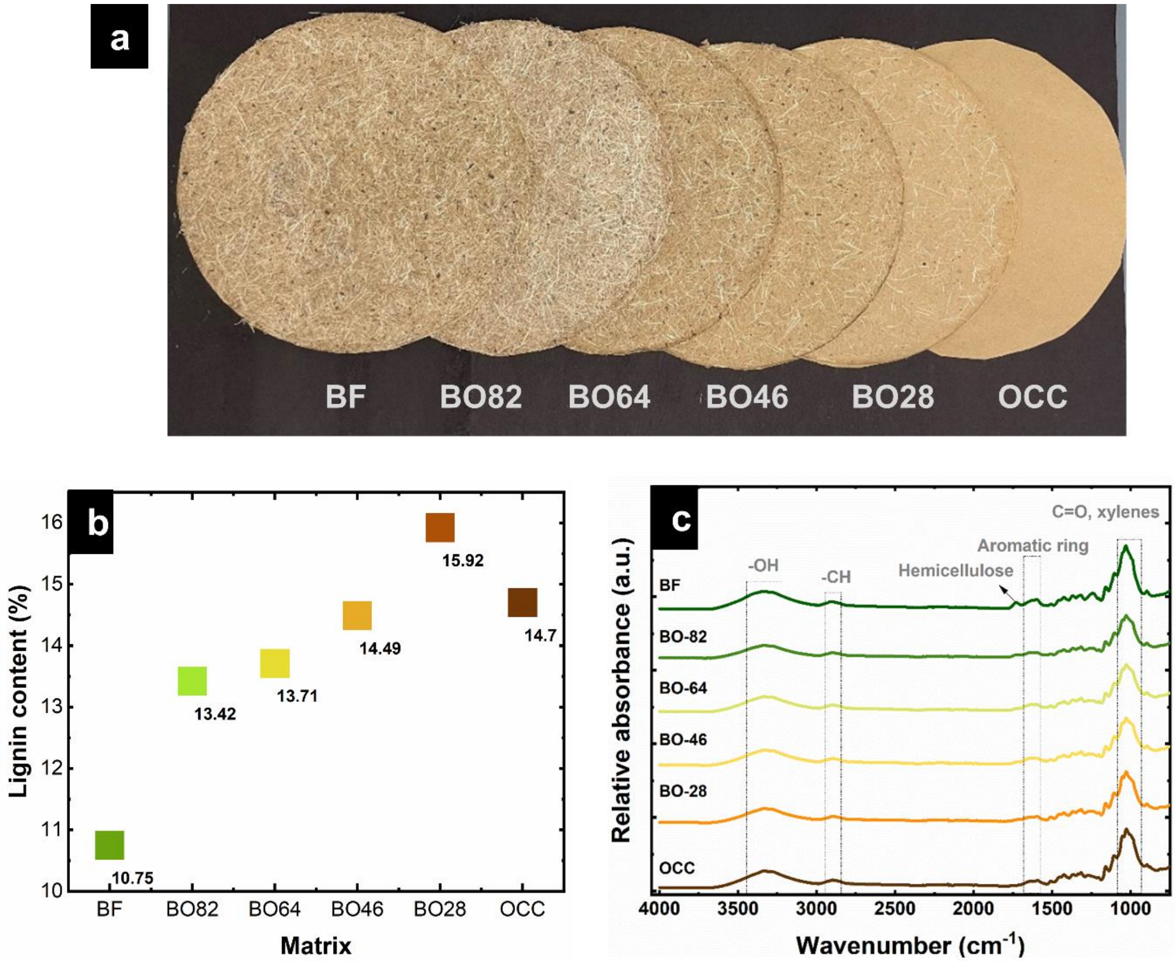

Supplementary Figure 1: Handsheets made from BF, OCC and hybrid fibers-Appearance & Composition. (a) Digital image showing dried handsheets prepared from banana fiber (BF), old corrugated cardboard boxes (OCC) and hybrid fibers. Plots showing (b) lignin content and (c) FTIR spectra of the matrices developed from BF, OCC and the hybrids.

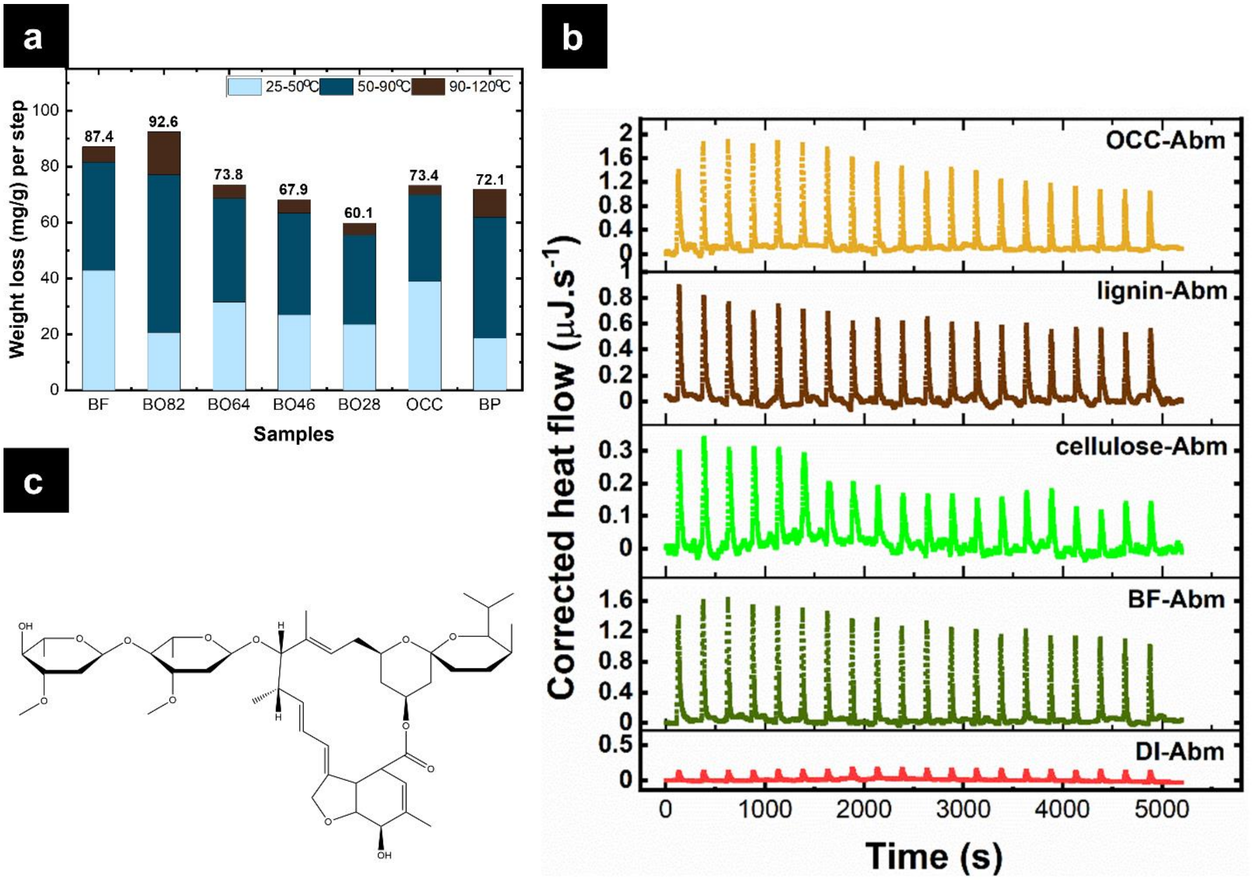

Supplementary Figure 2: Free & bound water content of matrices, ITC titrations & structure of abamectin. (a) Plot showing the weight loss (mg/g) for BF, OCC and hybrid papers per heating step between 25-50 ^{o}C, 50-90 ^{o}C and 90-120 ^{o}C to determine the free and bound water and volatiles content respectively. (b) ITC thermograms obtained after the titration of abamectin (Abm) solution with DI water, banana fiber (BF), cellulose and lignin at 25 ^{o}C. (c) The structural formula of Abm generate by ChemDraw.

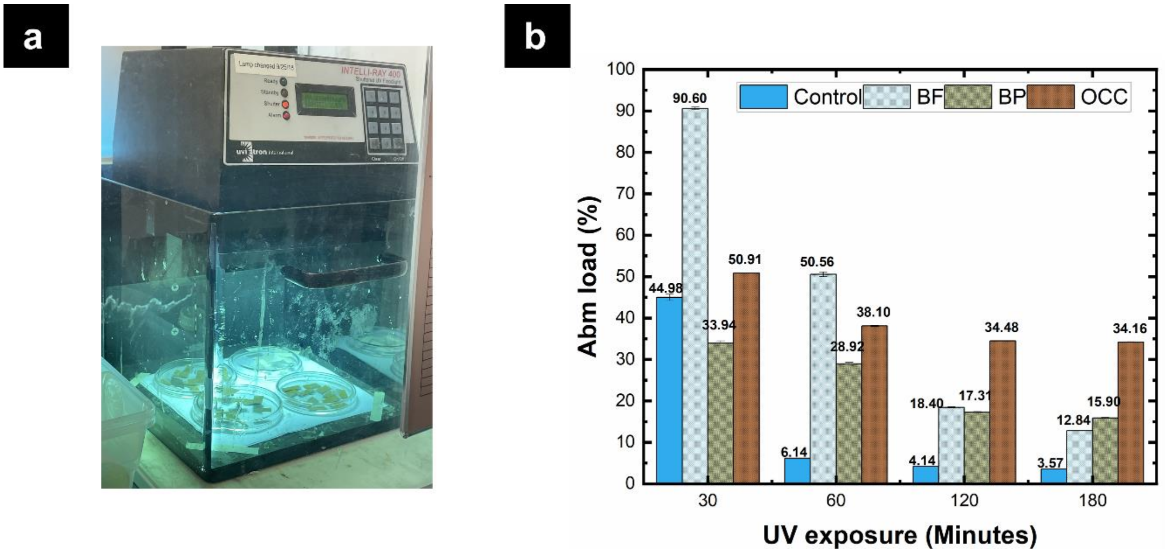

Supplementary Figure 3: Experiment setup and data showing delayed decomposition of Abm loaded on BP. (a) Experimental setup showing samples being exposed to UV light in a INTELLI-RAY 400 UV lamp. (b) Plot showing Abm load after UV exposure for 30, 60, 120 and 180 minutes (n=3). Control refers to Abm loaded on copy paper.

Supplementary Tables 1: Biodegradability and soil integrity studies. Tensile strength (MPa) of various samples before and after 7, 14 and 21 days in the soil.

Samples	BF:OCC (%)	Day 0	Day 7	Day 14	Day 21
**BF**	100:0	6.96	2.87	0.51	0.56
**BO82**	80:20	2.85	0.71	0.34	0.31
**BO64**	60:40	3.43	0.94	1.13	0.39
**BO46**	40:60	17.24	2.93	3.04	1.01
**BO28**	20:80	13.21	5.41	4.79	1.64
**OCC**	0:100	9.93	4.48	3.99	1.17
**BP**	80:20	5.59	2.42	2.31	0.05

Supplementary Tables 2: Physical attributes of various matrices. Comparative physical attributes of banana paper (BP) as compared to matrices developed from banana fiber (BF), OCC and hybrid fibers (BF:80% and OCC 20%) in terms of density, air resistance, burst index and weak/strong bonded Abm content.

**Samples**	**Density (kG/m^3)**	**Air resistance (gs)**	**Burst index $kPa.m^2/g$**	**Weakly bonded Abm**	**Strongly bonded Abm**
**BF**	372.7±20.7	2.06±0.92	3.83±0.602	32.5±0.32	67.5±0.32
**BO82**	243.9±33.2	1.28±0.33	3.27±0.506	13.2±0.40	91.8±0.40
**BP**	300.6±27.6	5.4±0.123	0.698±0.04	8.1±2.04	91.9±2.04
**OCC**	560.9±17.4	12.9±0.63	13.37±2.07	0	100±0

Supplementary Tables 3: Analysis of variance (ANOVA) data. Table showing ANOVA data for yam yield (kg/m^2), dry rot rating, and final population (Pf) of *Scutellonema bradys* per gram of yam peel combined for two growing seasons (2015-16) of yam in Benin. Data analysis consisted of one-way Analysis of Variance (ANOVA) for a randomized complete block design with three treatments and five replications. Combined analysis was done as for a factorial design with three treatments, five replications, 26 farms, and four years with no adjustments. All data analysis was accomplished using the General linear models procedure (PROC GLM) of PC/SAS software (SAS Institute, Cary, NC).

		**Yam yield (Kg/ m2)**		**Dry Rot**		**Pf *Scutelonema*/250 cm^3 soil**	
**Source**	**DF**	**F Value**	**Pr > F**	**F Value**	**Pr > F**	**F Value**	**Pr > F**
rep	4	1.37	0.2477	0.37	0.8269	1.39	0.2373
trt	2	179.49	<.0001	64.59	<.0001	1723.68	<.0001
farm	10	89.02	<.0001	10.82	<.0001	46.74	<.0001
Year	1	321.18	<.0001	1.01	0.3156	511.56	<.0001
trt*farm	20	7.43	<.0001	5.42	<.0001	19.16	<.0001
trt*Year	2	60.79	<.0001	0.46	0.6323	37.36	<.0001
farm*Year	4	11.51	<.0001	6.67	<.0001	25.09	<.0001
trt*farm*Year	8	5.77	<.0001	2.18	0.0309	8.48	<.0001

Supplementary Tables 4: Effect of W&P treatment on variation in nematode population during storage. *S.bradys* Rf in tuber peels and reproduction factor after 3- and 5-months storage in Save, Glazoue and Savalou counties from 2015-2018.

Year	Regions	Final nematode population (Pf) at harvest<br>No. of *S.bradys* /g of peels			Reproduction factor (Rf)					
		BP-Abm	BP	FP	After 3 months			After 5 months		
					BP-Abm	BP	FP	BP-Abm	BP	FP
**2015**	**Glazoué**	5.2±1.3	13.1±2.0	29.2±3.2	0.2±0.1	0.4±0.1	1.2±0.5			
	**Savè**	6.8±3.5	13.4±2.6	32.9±11.5	0.2±0.2	0.3±0.3	0.8±0.7			
**2016**	**Glazoué**	14.0±9.9	35.0±16.9	123.5±26.2	1.7±0.9	1.5±0.7	2.0±0.0			
	**Savè**	40.0±31.1	67.0±1.4	175.0±84.9	1.5±0.7	2.0±0.2	2.5±0.7			
**2017**	**Glazoué**	13.0±0.2	12.9±1.3	18.7±8.3	1.1±0.1	1.2±0.1	1.5±0.3	2.2±0.3	2.1±0.2	2.5±0.2
	**Savè**	12.0±0.3	36.5±3.2	49.9±13.2	1.3±0.3	1.8±0.3	2.8±1.5	2.1±0.3	2.7±0.2	3.2±1.3
	**Savalou**	10.1±2.5	17.6±3.8	17.6±3.8	0.9±0.1	1.4±0.2	1.7±0.1	1.6±0.1	2.3±0.2	2.5±0.2
**2018**	**Savalou**	5.2±1.8	6.6±2.8	11.4±4.6	1.9±0.2	2.1±0.4	2.0±0.5	2.7±0.2	2.8±0.5	2.8±0.5

Supplementary Tables 5: Soil attributes of various communities. The texture (% sand, silt, clay) and pH of soils for various communities that were used in the field trials.

Commune	Code	Soil texture			
		**% sand**	**% silt**	**% clay**	**pH**
Glazoué	GDH	77.1	13.0	9.2	6.7
Glazoué	GHC	75.9	11	12	6.2
Glazoué	GAA	76.4	13.7	9.3	6.5
Glazoué	GYI	78.5	11.3	9.7	6.2
Glazoué	GBV	71.9	14.1	13.3	5.9
Savè	STA	81.9	8.6	9.1	5.8
Savè	SCK	79	10.9	9.4	5.5
Savè	SSA	81	7.7	10.6	5.4
Savè	SES	78.5	10.3	10.6	5.6
Savè	SGD	78.9	11.2	9.4	5.4

Reference

1. Khalil, M. S. and Darwesh, D. Avermectins: The promising solution to control plant parasitic nematodes. Journal of Plant Science and Phytopathology 81-85 (2019).
