# Supplementary material for: Plant-biomass-based hybrid seed wraps mitigate yield and post-harvest losses among smallholder farmers in sub-Saharan Africa
Source: Nat Food. 2023 Feb 16;4(2):148–59. doi: 10.1038/s43016-023-00695-z (PMC10154224; doi:10.1038/s43016-023-00695-z)
Supplement: Supplementary file 2 — Reporting Summary [file 43016_2023_695_MOESM2_ESM.pdf]

nature portfolio

Corresponding author(s): Saad A. Khan, Charles H. Opperman

Last updated by author(s): Jan 5, 2023

Reporting Summary

Nature Portfolio wishes to improve the reproducibility of the work that we publish. This form provides structure for consistency and transparency in reporting. For further information on Nature Portfolio policies, see our Editorial Policies and the Editorial Policy Checklist.

Statistics

For all statistical analyses, confirm that the following items are present in the figure legend, table legend, main text, or Methods section.

n/a	Confirmed	
☐	☒	The exact sample size (*n*) for each experimental group/condition, given as a discrete number and unit of measurement
☐	☒	A statement on whether measurements were taken from distinct samples or whether the same sample was measured repeatedly
☐	☒	The statistical test(s) used AND whether they are one- or two-sided<br>*Only common tests should be described solely by name; describe more complex techniques in the Methods section.*
☒	☐	A description of all covariates tested
☒	☐	A description of any assumptions or corrections, such as tests of normality and adjustment for multiple comparisons
☐	☒	A full description of the statistical parameters including central tendency (e.g. means) or other basic estimates (e.g. regression coefficient) AND variation (e.g. standard deviation) or associated estimates of uncertainty (e.g. confidence intervals)
☐	☒	For null hypothesis testing, the test statistic (e.g. *F*, *t*, *r*) with confidence intervals, effect sizes, degrees of freedom and *P* value noted<br>*Give P values as exact values whenever suitable.*
☒	☐	For Bayesian analysis, information on the choice of priors and Markov chain Monte Carlo settings
☒	☐	For hierarchical and complex designs, identification of the appropriate level for tests and full reporting of outcomes
☒	☐	Estimates of effect sizes (e.g. Cohen's *d*, Pearson's *r*), indicating how they were calculated

Our web collection on statistics for biologists contains articles on many of the points above.

Software and code

Policy information about availability of computer code

Data collection	OMNIC software for FTIR; TA instruments SDT software for TGA; TA instruments Trios software for DMA,
Data analysis	TA instruments Nanoanalyzer software Version 3.11.0 for ITC; Universal analysis 2000 Version 4.5A for Windows/XP/Vista for TGA; SAS version 9.4. SAS Institute Inc. The SAS System for Windows, Version 9.4; SAS Institute Inc.: Cary, NC, USA, 2013. Microsoft Excel for Mac version 16.61. Chemdraw Professional 20.1.0.110 for chemical structures

For manuscripts utilizing custom algorithms or software that are central to the research but not yet described in published literature, software must be made available to editors and reviewers. We strongly encourage code deposition in a community repository (e.g. GitHub). See the Nature Portfolio guidelines for submitting code & software for further information.

Data

Policy information about availability of data

All manuscripts must include a data availability statement. This statement should provide the following information, where applicable:

- Accession codes, unique identifiers, or web links for publicly available datasets
- A description of any restrictions on data availability
- For clinical datasets or third party data, please ensure that the statement adheres to our policy

All data generated or analyzed during this study are included in this published article and its supplementary information files

March 2021

Human research participants

Policy information about studies involving human research participants and Sex and Gender in Research.

Reporting on sex and gender	*Use the terms sex (biological attribute) and gender (shaped by social and cultural circumstances) carefully in order to avoid confusing both terms. Indicate if findings apply to only one sex or gender; describe whether sex and gender were considered in study design whether sex and/or gender was determined based on self-reporting or assigned and methods used. Provide in the source data disaggregated sex and gender data where this information has been collected, and consent has been obtained for sharing of individual-level data; provide overall numbers in this Reporting Summary. Please state if this information has not been collected. Report sex- and gender-based analyses where performed, justify reasons for lack of sex- and gender-based analysis.*
Population characteristics	*Describe the covariate-relevant population characteristics of the human research participants (e.g. age, genotypic information, past and current diagnosis and treatment categories). If you filled out the behavioural & social sciences study design questions and have nothing to add here, write "See above."*
Recruitment	*Describe how participants were recruited. Outline any potential self-selection bias or other biases that may be present and how these are likely to impact results.*
Ethics oversight	*Identify the organization(s) that approved the study protocol.*

Note that full information on the approval of the study protocol must also be provided in the manuscript.

Field-specific reporting

Please select the one below that is the best fit for your research. If you are not sure, read the appropriate sections before making your selection.

☒ Life sciences ☐ Behavioural & social sciences ☐ Ecological, evolutionary & environmental sciences

For a reference copy of the document with all sections, see nature.com/documents/nr-reporting-summary-flat.pdf

Life sciences study design

All studies must disclose on these points even when the disclosure is negative.

Sample size	Paper handsheets: 10 handsheets were produced for each sample<br>Seed wrap: 10, 000 seed wraps (6*8 inches2) were produced from 7500 feet of paper roll.<br>Each field trial was arranged in a randomized complete block design with five replicates and three treatments. Individual plots, spaced 2meters apart, accommodated four rows of 6 mounds. After the mounds were prepared for planting and labeled following the treatments, soil samples were collected to estimate pre-planting nematode population densities. Four soil cores were removed per plot from 5 to30 cm depth using a hand trowel, following a zig-zag sampling pattern. Soil cores from the same plot were combined and thoroughly mixed before a250 cm^3 composite sample was removed for nematode extraction using the centrifugation technique. Each mound was planted with single seed yam.Fields harbored an initial nematode density of at least 500 nematodes per 250 cm^3 soil.<br>Post-harvest studies: sample peels (outer cortex) were removed from a 5×5 cm2 area on four sides of each of three tubers, to determine tuber nematode population densities at harvest and after 3- and 5-month storage. Tuber peels from the same plot were then thoroughly mixed, and 25 g peels were removed for nematode extraction using the centrifugation technique. For soil nematodes, soil sub-samples were collected from the tuber zones in the middle mounds, and a composite soil sample of 250 cm3 was removed per plot and processed for population estimation.<br>Soil and root sampling for nematodes employed standard protocols that have been robustly applied for over 50 years for numerous crops. Sample size for yam was chosen to closely replicate farmer's practices and was limited by the size of the plots we were able to deploy but were representative of local yam production practices.
Data exclusions	No data were excluded during the characterization of banana paper.<br>No data were excluded from the analyses in the field trials since none of the plants selected for individual monitoring in each plot after emergence die at the end of the experiment.
Replication	Seed wraps: To characterize burst index, density and air resistance of banana paper handsheets and paper roll, we used 10 replicates in each case following standard TAPPI protocols. 3 replicates were used to determine mechanical strength and integrity via DMA analysis of each sample. 3 replicates were used in each case to determine bioavailability, binding analysis and UV-resistance of respective samples.<br>Paper for the field trials: Each year, the paper for the seed wraps was prepared following the same protocol and its key attributes were evaluated to replicate those of the previous years' prior to use in the field trials.<br>Field trials: The field study was undertaken from 2015 to 2018 in Guinea-Sudan transition zone of Benin (Centre of Benin, West Africa) in three districts. Field trials were replicated for three consecutive years in a total of 16 farmers' fields at Glazoué, and Savè, and for two consecutive years at Savalou. The trials were replicated in the same field from 2016-2017 in Glazoué and Savè, and from 2017-2018 inSavalou .Seed yams for the first year of trials were produced in nematode-free soil using miniset technique. For the following years, seed yams were purchased from farmers who had been previously trained for the production of nematode-free seed yams.<br>Planting: Planting occurred at the beginning of the first rainy season (8-11 June, 2015; 13-16 May, 2016; 26 April-29 May, 2017; 8 May 2018),and tubers were harvested 7-8 months later when vines were completely dried. In each season, trials were replicated in multiple different farmers' fields (2015: 6 farms; 2016: 4 farms; 2017: 10 farms; 2018: 6 farms), and trials were repeated for four seasons. In total,

	trials were established in 16 different farmers' fields (10 farms for multiple years) with a similar history of yam cultivation during 2015 thru 2018. All attempts at replication were successful. Typical farmer's practice (FP) is to plant the yam seed piece in the mound with no further inputs. Farmer's practices vary among farms, but weed control is via manual cultivation when it does occur, and no fertilizer is added to the mounds. No insect or disease control agrochemical applications are employed. Yam cultivation is very low input and labor intensive, with crop management being performed manually.The site of the storability experiment is located in the sub-humid savannah region with a sub-equatorial climate characterized by two wet seasons from mid-March to mid-July and mid-September to mid-November, alternating with two dry seasons. The annual average rainfall is between 1000 and 1200 mm and the yearly mean temperature between 25 and 30 oC. Data analysis for each site consisted of Analysis of Variance (ANOVA) for a randomized complete block design with three treatments (FP, BP,and BP-Abm) and five replications. The yearly results were combined to determine the differences between years.
Randomization	Field trials were fully randomized in the open field, due to the homogeneous infestation of the soil with plant-parasitic nematodes, the lack ofinclination of the field and the absence of physical elements (e.g. trees, rocks, shelters) in the experimental area,. Plants were distributedusing a randomized complete block design.
Blinding	Blinding was not relevant to field trials of this nature. Field trials: In each location, we gave every treatment and plot number a unique random code (e.g. BP-Abm would be GBV 101, etc.) that we entered into an excel file. Plants within each replication plot were labelled as GBV 101.1, GBV 101.2, etc., until the end of the season and the data collection process. Laboratory supporting staff worked using these codes.

Reporting for specific materials, systems and methods

We require information from authors about some types of materials, experimental systems and methods used in many studies. Here, indicate whether each material, system or method listed is relevant to your study. If you are not sure if a list item applies to your research, read the appropriate section before selecting a response.

Materials & experimental systems

n/a	Involved in the study
☒	☐ Antibodies
☒	☐ Eukaryotic cell lines
☒	☐ Palaeontology and archaeology
☐	☒ Animals and other organisms
☒	☐ Clinical data
☒	☐ Dual use research of concern

Methods

n/a	Involved in the study
☒	☐ ChIP-seq
☒	☐ Flow cytometry
☒	☐ MRI-based neuroimaging

Animals and other research organisms

Policy information about studies involving animals; ARRIVE guidelines recommended for reporting animal research, and Sex and Gender in Research

Laboratory animals	Caenorhabditis elegans strain N2, hermaphrodite, +/- 3.5 day lifecycle
Wild animals	It is likely that numerous different species of plant-parasitic nematodes were isolated during soil sampling for these experiments, but the predominant species we recovered was Scutellonema bradys, a pernicious pest invertebrate. These microscopic nematodes were collected in soil and root/peel samples, were extracted by standard techniques, and disposed of down the sink drain. Prior to extraction the samples were held in refrigerated conditions.
Reporting on sex	Sex was not considered during study design
Field-collected samples	It is likely that numerous different species of plant-parasitic nematodes were isolated during soil sampling for these experiments, but the predominant species we recovered was Scutellonema bradys, a pernicious pest invertebrate. These microscopic nematodes were collected in soil and root/peel samples, were extracted by standard techniques, and disposed of down the sink drain. Prior to extraction the samples were held in refrigerated conditions.
Ethics oversight	No ethical guidance required. C. elegans is a standard laboratory invertebrate and plant-parasitic nematodes are pernicious pests. There are no ethical or regulatory concerns with working with either organism.

Note that full information on the approval of the study protocol must also be provided in the manuscript.
